# First person – Varsha N. Tamilkumar

**DOI:** 10.1242/bio.062243

**Published:** 2025-09-26

## Abstract

First Person is a series of interviews with the first authors of a selection of papers published in Biology Open, helping researchers promote themselves alongside their papers. Varsha N. Tamilkumar is first author on ‘ Epithelial fusion is mediated by a partial epithelial–mesenchymal transition’, published in BiO. Varsha conducted the research described in this article while a PhD student in Professor Raj Ladher's lab at NCBS-TIFR, Bengaluru, India. She is now a postdoc in the lab of Professor Hiroshi Hamada at NCBS-TIFR, Bengaluru, India, investigating early embryonic development, specifically pattern formation and morphogenesis.


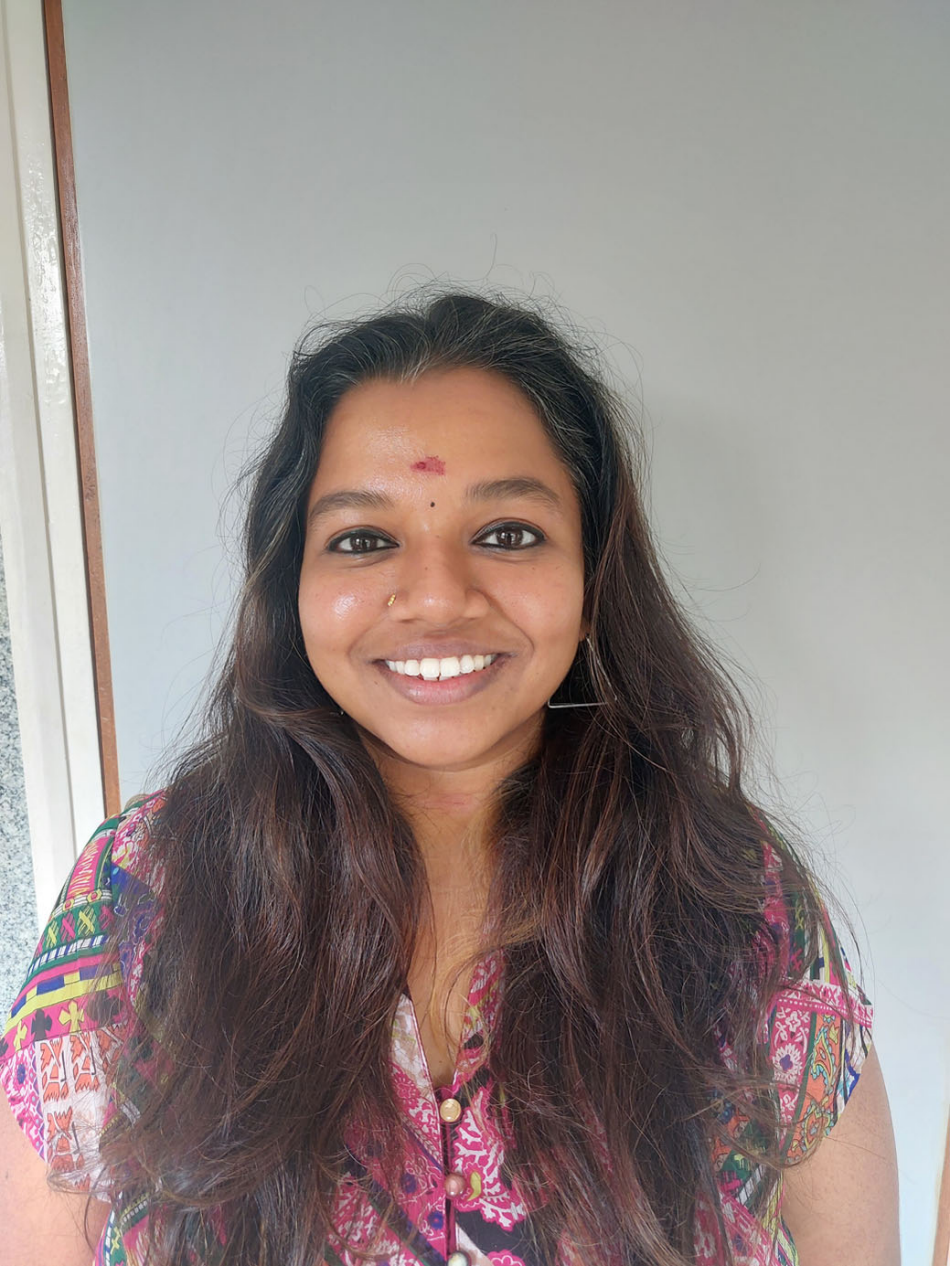


**Varsha N. Tamilkumar**

**Describe your scientific journey and your current research focus**

I was trained as an engineer in Industrial Biotechnology before I joined a chemical ecology lab. This is where I was fascinated by how a moth larva could eat toxic substances throughout its larval stage and still develop into a beautiful adult. This got me interested in the robustness in embryonic development and I joined Professor Raj Ladher's lab. His lab works on inner ear development using chick and mouse embryos. I did my PhD in early inner ear development, specifically how epithelial fusion helps in the closure of the inner ear primordia, otic vesicle. We have observed that the fusing cells undergo a partial epithelial to mesenchymal transition during the fusion.

**Who or what inspired you to become a scientist?**

My first inspiration comes from two movies, ‘Honey, I shrunk the kids’ and ‘Honey, I blew up the kid’. Although it was about a robotic engineer, it inspired me to run wild with the scope of what scientific curiosity can unearth and be the best in whatever I decided to become. My parents never failed to encourage my life choices. Their patience in answering my curious questions kept me engaged in academia. All of my teachers had great expectations of me, which pushed me to strive for better things.

My first inspiration comes from two movies, ‘Honey, I shrunk the kids’ and ‘Honey, I blew up the kid’.

**How would you explain the main finding of your paper?**

All of us have seen our open wounds gradually shrink and then close, this process is wound healing. The closing of the skin is very similar to other processes that happen during embryonic development. Multicellular organisms have two types of cells: a rigid epithelial cell and a fluid mesenchymal cell. In normal development, the cells can transform from one type to another, reversibly. Epithelial cells are part of a continuous system, for example, the skin. As seen in wound healing, epithelial cells from two sides of the wound come together and fuse to close a gap. The inner ear development has a similar process, when epithelial cells from opposite sides come together and close to become the inner ear. We found that the epithelial cells that fuse are not completely rigid and adapt a reversible, fluid-like trait that helps in the fusion.

We found that the epithelial cells that fuse are not completely rigid and adapt a reversible, fluid-like trait that helps in the fusion.

**What are the potential implications of this finding for your field of research?**

Partial epithelial–mesenchymal transition has been studied in cancer cell lines extensively. In our study, we characterize these cells in normal embryonic development, including cell shape changes, junction complex localization, and transcriptomic signature. This might help us to understand other fusion processes, including neural tube closure and palatal shelf closure. These processes often get disturbed and manifest as birth defects of varying severity. Our study has contributed to understanding this process, which might help us in reducing the frequency of these birth defects.

**Figure:**
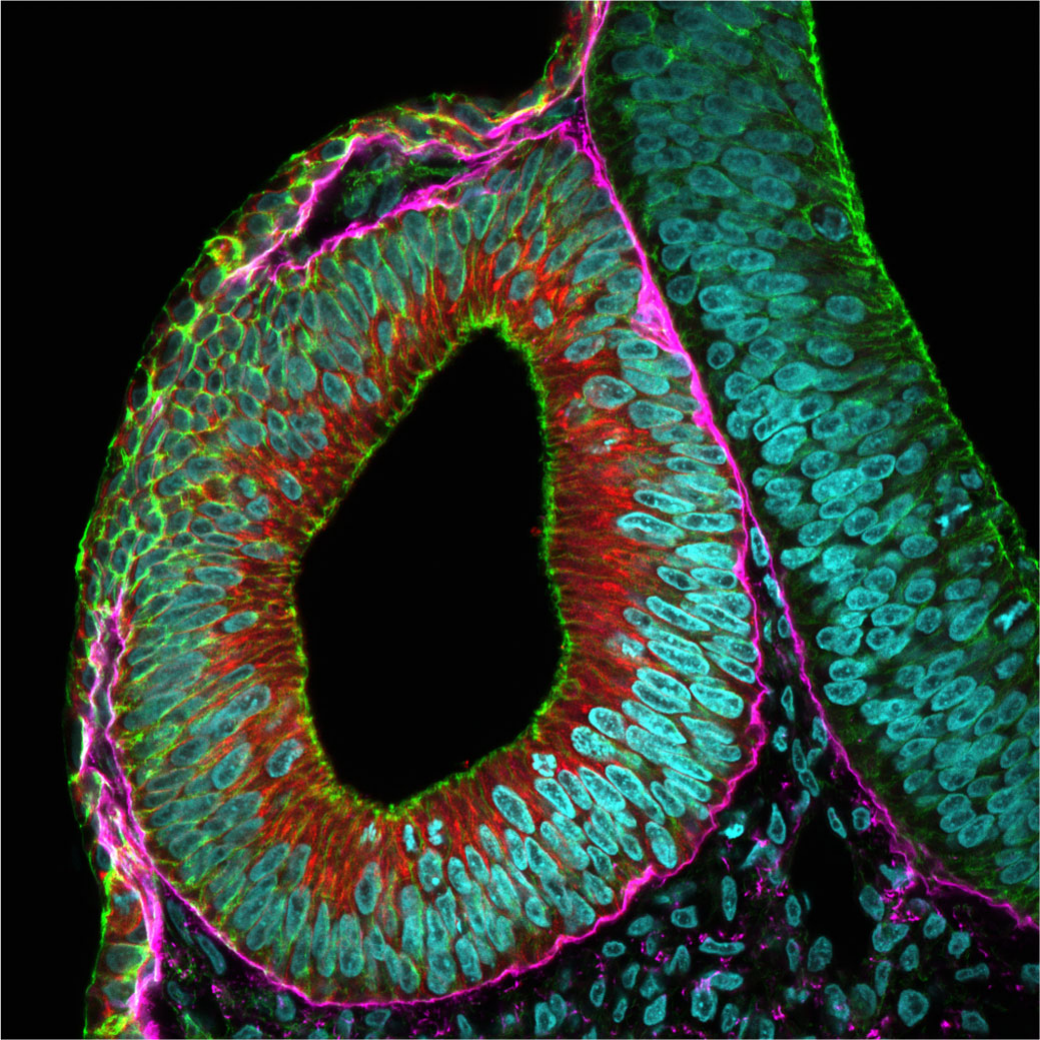
A closing otic vesicle in continuation with the surface ectoderm, which will eventually segregate from the surface ectoderm and be internalized.

**Which part of this research project was the most rewarding?**

There were two results that were the most rewarding; the first was the transcriptomics data, when we saw a clear segregation of the three tissue types. It took me a while to dissect the small tissue with tungsten needles, and I was unable to check for any cross contamination. We proceeded anyway with the bulk-mRNA seq, and got good quality reads. The second was the phenotype we got with the CRISPR-Cas9 based perturbation.

**What do you enjoy most about being an early-career researcher?**

I have been fortunate with my PIs. They have always given me the freedom to work on my ideas, while guiding me whenever necessary. Having the right mentor makes a huge difference in the quality of work we do, and my PhD guide and postdoc guide are incredibly supportive. I absolutely love being able to do a variety of experiments without having to do any bureaucratic work or having to write for grants. I have the luxury of only focusing on my work and not having to worry about anything else.

**What piece of advice would you give to the next generation of researchers?**

It is important to design straightforward experiments that will yield non-confounding results. It is difficult to stay away from new techniques that are being developed, but what we often fail to evaluate is the utility of this ‘latest’ technology in our study. The major thing that should drive the scientist in you is curiosity and an awe for nature.

**What's next for you?**

I am currently a postdoc in Hiroshi Hamada's lab in NCBS, Bengaluru. We work on left–right asymmetry in chick and snail embryos. I hope to continue in academia after my postdoc as well.
